# What is the economic burden of delayed axial spondyloarthritis diagnosis in the UK?

**DOI:** 10.1093/rheumatology/keaf226

**Published:** 2025-04-25

**Authors:** Fernando Zanghelini, Georgios Xydopoulos, Stephanie Howard Wilsher, Oyewumi Afolabi, Dale Webb, Joe Eddison, Thomas A Ingram, Clare Clark, Jill Hamilton, Raj Sengupta, Karl Gaffney, Richard Fordham

**Affiliations:** Health Economics Consulting, Norwich Medical School, University of East Anglia, Norwich, UK; Health Economics Consulting, Norwich Medical School, University of East Anglia, Norwich, UK; Health Economics Consulting, Norwich Medical School, University of East Anglia, Norwich, UK; Health Economics Consulting, Norwich Medical School, University of East Anglia, Norwich, UK; National Axial Spondyloarthritis Society (NASS), London, UK; National Axial Spondyloarthritis Society (NASS), London, UK; National Axial Spondyloarthritis Society (NASS), London, UK; National Axial Spondyloarthritis Society (NASS), London, UK; National Axial Spondyloarthritis Society (NASS), London, UK; Rheumatology, Royal National Hospital for Rheumatic Diseases, Bath, UK; Rheumatology, Norfolk & Norwich University Hospitals NHS Foundation Trust, Norwich, UK; Health Economics Consulting, Norwich Medical School, University of East Anglia, Norwich, UK

**Keywords:** axial spondyloarthritis, ankylosing spondylitis, economic evaluation, cost, delayed diagnosis, burden

## Abstract

**Objective:**

The objective of this study was to develop an economic model to determine the annual cost of delayed axial SpA diagnosis in the UK, adopting both National Health Service (NHS) and societal perspectives.

**Methods:**

We developed a Markov economic model to estimate the costs of delayed axial SpA diagnosis in the UK. Model parameters were sourced from a 2016 National Axial Spondyloarthritis Society patient survey, anonymized patient-level data, the published literature, and expert opinion. A literature-defined, mixed cohort (64% male) of people assumed to have axial SpA, whose age at symptom onset was 26 years, were targeted. To assess the robustness of the results, base case and probabilistic sensitivity analyses were performed.

**Results:**

In a simulated cohort of 1000 patients, with a mean time to diagnosis of 8.5 years, we estimate the cumulative costs of delayed diagnosis per person living with axial SpA to be £193 512 (95% CI: 108 770–306 789). The costs were led by productivity losses (65.1%) and out-of-pocket expenses (31.3%). The total annual cost resulting from delayed axial SpA diagnosis in the UK has been estimated at £3.1 billion and £12.5 billion, based on a prevalence of 0.3% (Assessment of SpondyloArthritis international Society classification criteria) and 1.2% (European Spondyloarthropathy Study Group classification criteria), respectively.

**Conclusion:**

Delayed axial SpA diagnosis carries high costs for society due to productivity losses. Early diagnosis and treatment could offer significant benefits to the patient and potentially reduce productivity losses; however, future research is needed to evaluate the long-term health economic impact.

Rheumatology key messages

- The estimated cumulative UK cost of delayed diagnosis per person living with axial SpA is £193 512.
- Costs of delayed axial SpA diagnosis (8.5 years) are substantial for the patient and society.
- Reducing the time to diagnosis in axial SpA could impact the societal economic burden.

## Introduction

Axial spondyloarthritis (axial SpA) is an immune-mediated inflammatory disease that predominantly affects the axial skeleton (SI joints and spine) and is typified by chronic back pain, peripheral musculoskeletal (MSK) manifestations (arthritis, enthesitis and dactylitis), and extra-musculoskeletal manifestations (EMMs; acute anterior uveitis, psoriasis and IBD) [1, 2]. The term axial SpA encompasses individuals with definitive sacroiliitis on plain radiographs (radiographic axial SpA; formerly known as AS) and individuals without definitive structural damage (non-radiographic axial SpA) [1, 2]. Symptoms usually begin in early adulthood, with the median age of onset being 26 years and the majority (92%) experiencing symptoms prior to the age of 45 years [3]. Recent reports from a multi-country, prospective, observational study suggest that only 16% of patients with non-radiographic axial SpA progress to radiographic axial SpA within 5 years [4].

Typical clinical features of axial SpA include chronic back pain and stiffness; however, fatigue, sleep disturbance, negative affect, and psychological distress are also frequent [5]. As a result, people living with axial SpA may experience reduced physical function, poor work outcomes [6] and worse quality of life [7]. The prevalence of axial SpA in the UK general adult primary care population is estimated to be between 0.3% (Assessment of SpondyloArthritis international Society [ASAS] classification criteria) and 1.2% (European Spondyloarthropathy Study Group [ESSG] classification criteria) [8].

The mean time to diagnosis in axial SpA in the UK is 8.5 years [9], which is higher than the worldwide mean estimates for axial SpA (6.7 years) and PsA (2.6 years) [10]. The reasons for diagnostic delay are complex and include: poor health-care professional (HCP) awareness; axial SpA representing an uncommon cause of chronic lower back pain; the existence of outdated misconceptions (e.g. axial SpA as a male disease); a lack of diagnostic criteria; misleading biomarkers; and, imaging difficulties [11]. Identifying the characteristics of individuals most at risk of diagnostic delay remains an important issue, with literature to date lacking consensus [10, 12]. Delay in the diagnosis of axial SpA may result in considerable clinical, humanistic and economic burden [13]. For example, individuals with a lengthy time to diagnosis may have worse physical function, more structural damage, a greater likelihood of depression and work disability, and higher direct and indirect health-care costs compared with those with an earlier diagnosis [13].

The economic burden of axial SpA remains a significant issue and is largely defined by health-care costs (e.g. health-care visits and medical tests), societal costs (productivity losses in paid work) and patient costs (e.g. out-of-pocket expenses). A recent study in Spain found that half of patients with axial SpA used ≥25 health-care resources (i.e. health-care visits, emergency visits, hospital admissions and medical tests) over a 12-month period [14]. Further, in a prospective cohort study of 1188 individuals living with axial SpA in Great Britian, 19% reported absenteeism, and 79% reported presenteeism, in the past week owing to their axial SpA [6]. In another UK-based cohort study, individuals with radiographic axial SpA (*n* = 570) reported missing 3.5% of their work time on average and a 21.6% impairment of working level, which in 2014, represented an average estimated cost of £869 due to absenteeism and £7241 due to presenteeism per year per working radiographic axial SpA patient [15]. The estimated total cost of radiographic axial SpA in the UK (in 2014) was £19 016 per patient per year, when including general practice attendance, administration and hospital costs, out-of-pocket expenses, and productivity losses [15]. Indeed, multiple international studies have demonstrated the substantial cost of axial SpA [16–21], but few have estimated the economic burden of delayed diagnosis.

Understanding the economic burden of delayed diagnosis is of primary concern, with many individuals seeing multiple HCPs [22] or receiving various therapies, prior to diagnosis [23]. Further, the amplified costs associated with increased disease burden and impaired function [15] are likely to compound the economic burden of diagnostic delay, as individuals are denied timely access to advanced therapies. Mennini *et al.* recently explored the economic impact of diagnostic delay in Italy, although the model only considered direct costs for specialist health-care services and pharmacological treatments before a diagnosis of SpA [24]. These data cannot be extrapolated to the UK, as costs and health-care systems vary between countries. No studies have explored the direct and indirect costs of delayed axial SpA diagnosis in the UK. Thus, the objective of this study was to develop an economic model to determine the annual cost of delayed diagnosis of axial SpA, adopting the perspective of the health-care system in the UK [National Health Service (NHS) England] and the societal perspective.

## Methods

### Study design

We developed a Markov economic model to understand and estimate the economic impact of delayed axial SpA diagnosis in the UK. As such, the model estimated the cumulative costs of an 8.5-year time to diagnosis, focusing on costs accrued up to the point of diagnosis. We assumed that after diagnosis the patient will receive effective treatment, and their condition will be kept under control.

The Markov model is an analytical framework commonly used to represent stochastic processes—random processes that change over time. Markov models are frequently used in economic evaluations of health-care interventions and are suited to modelling chronic disease. The disease is defined in terms of mutually exclusive health states, and individuals can move or transition between states (represented as transition probabilities) over a discrete period (Markov cycle). Estimated costs and health outcomes can be attached to states and transitions and then modelled for a patient cohort over successive cycles to estimate long-term costs and outcomes associated with a disease or intervention [25].

The model was constructed using Microsoft Excel and the analysis was reported according to the Consolidated Health Economic Evaluation Reporting Standards (CHEERS) statement for transparency [26]. For Patient and Public Involvement and Engagement (PPIE), stakeholders/experts by experience were interviewed for their insight into axial SpA and to help develop the economic model.

### Decision model structure

The Markov model captured the resources used and costs related to the diagnosis, management of symptoms, and impacts of axial SpA until the diagnosis of the disease and initiation of treatment (costs after a diagnosis were excluded). Assuming a ‘Gold Standard’ time to diagnosis of 1 year [27], the sum of costs in each consecutive cycle was estimated.

To develop the model and understand the economic impact of diagnostic delay, semi-structured interviews with key stakeholders [one general practitioner (GP), one GP/clinical commissioner, one osteopath and four people living with axial SpA] and a focus group with clinical stakeholders (two chiropractors, two physiotherapists, two first-contact practitioners, one GP, one rheumatology consultant, and one osteopath) were undertaken. This PPIE was used to check that appropriate variables and parameters were added to the model.

**Figure 1.**
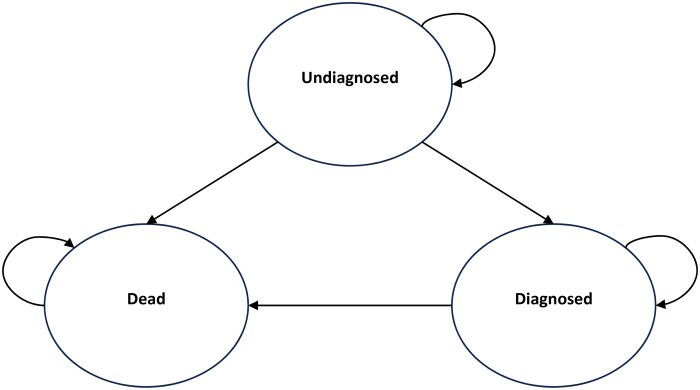
Markov economic model

Fig. 1 presents the structure of the Markov economic model. In summary, the model has three health states. People start in the model at the undiagnosed health state and remain in that health state until they are diagnosed with axial SpA or die for any reason. Once people are diagnosed, they move into the diagnosed health state and remain in this health state, receiving treatment until they move into the dead health state.

The long-term costs associated with an axial SpA diagnosis were not modeled, because they were outside the scope of the research. The model does not distinguish between radiographic axial SpA and non-radiographic axial SpA, as they share similar clinical features and most likely lie on a spectrum of the same disease entity [28].

### Time horizon

The economic evaluation estimated costs over a lifetime horizon to accurately calculate resources used and costs related to delay in diagnosis of axial SpA. Furthermore, the model followed patients in 3-month cycles to capture any resource utilization and cost related to diagnosis at the predefined stage. The model had a lifetime horizon, as diagnostic delay may span over decades and have an impact on the costs incurred (over a lifetime) [29].

### Study perspective

Economic analysis was carried out using the costs collected from the NHS England perspective (to estimate direct medical costs), the societal perspective (to estimate productivity losses in paid work due to absenteeism, presenteeism and staff turnover) and the patient perspective (to estimate out-of-pocket expenses). All costs were reported in British pounds for the years 2021–22. Costs were discounted at the standard annual rate of 3.5%, adhering to the NICE Guidelines Manual [30].

### Model input parameters

#### Study population

To accurately calculate the cost of delayed diagnosis of axial SpA, the modelled population comprised people that we considered a priori to have axial SpA. We modelled the patient journey towards diagnosis based on the transition probabilities gathered from diverse sources. Results from the online NASS *State of the Nation Survey* (2016), which was distributed by NASS to people living with axial SpA in the UK [31], were used as inputs for the economic modelling. Anonymized patient-level data from the Norfolk and Norwich University Hospital (NNUH) and the Royal National Hospital for Rheumatic Diseases (RNHRD) on the patient journey to diagnosis were also utilized [9]. Secondary data sources such as the NICE NG65 model [32] and evidence from the extant literature and expert opinion were used to generate data input. Further details on the input parameters used in the model are described below and in Supplementary Table S1, available at *Rheumatology* online.

The target population comprised people whose age at symptom onset was 26 years [3]. The percentage of male patients for the mixed cohort calculations was 64%, as defined in the NICE NG65 model [32]. The standardized mortality rate for male and female patients was set to 1.630 and 1.380, respectively, exposing patients to a lengthy disease burden [33]. Diagnostic sensitivity and specificity were obtained from the study developed by Van Hoeven *et al.* [34].

#### Formal care

Formal care use and associated costs included an estimate of annual visits to GP and physiotherapist services. The probability of comorbidities appearing within 3 months was also estimated based on an 8.5-year time to diagnosis, an assumed prevalence of specific comorbidities [35, 36] and experts’ opinions. Costs were taken from the latest Personal Social Services Research Unit (PSSRU) cost publication [37].

All rates and probabilities were adjusted based on formulas to refer to the correct time frame. All costs were inflated to 2021 values using the NHS Cost Inflation Index (NHSCII) [37] and the Treasury Green Book on the social discount rate [38]. Further details can be seen in Supplementary Table S1, available at *Rheumatology* online.

#### Out-of-pocket expenses

The frequency of visits to chiropractic and osteopathic services, and the use of over-the-counter (OTC) medications, were assumed as reported in the NASS *State of the Nation Survey* (2016) [31]. In this survey, respondents with axial SpA were asked to recall their usage of specific medications and visits to a HCP in the last 12 months.

Based on discussions with stakeholders (patients and rheumatologists), it was assumed that individuals would self-manage pain with OTC medications. We assumed the maximum dosage allowed in the British National Formulary to estimate the monthly cost of these medications [39]. Prices for OTC medicines were obtained online from Boots Pharmacy. Patients who often purchase multiple medications monthly may obtain a prescription from their GP (regardless of their availability OTC) to control monthly costs. Therefore, the cost of the medicine was estimated so that the monthly cost did not exceed the monthly NHS prescription rate of £9.35. Our calculation also considered the availability of NHS prescription pre-payment certificates [40], so that the annual cost of the medicine did not exceed £108.10 per person.

We estimated the cost of travel to and from general, physiotherapy, osteopath, and chiropractor practices. The average travelling distance to a general practice in the UK was acquired from the Department of Transport journey time statistics [41]. The average taxi price for this travelling distance was used to estimate cost. A car (or taxi) was the presumed mode of transportation, based on the assumption that individuals might opt for a comfortable means of transportation, especially if they are experiencing some degree of pain. The same assumption was utilized to estimate the travel costs to and from osteopath and chiropractic visits. The number of GP visits during the period of diagnostic delay and the costs of non-prescribed exercise per person were based on research by Cooksey *et al.* [15]. Further details on the out-of-pocket expenses and their respective sources are described in Supplementary Table S1, available at *Rheumatology* online.

#### Productivity losses

The cost of productivity losses (absenteeism, presenteeism and staff turnover) were sourced from the Deloitte report and based on estimations for public and private sector employees [42]. Calculations were adjusted by the self-reported data on employment status and impact of disease on employment (e.g. probabilities of experiencing productivity loss) [31], and the gross median wage per hour per gender [43]. The costs and percentages of early retirement and of people requiring unpaid care assistance due to axial SpA were also included [15, 44] (see Supplementary Table S1, available at *Rheumatology* online).

#### Prevalence of axial SpA

To calculate the national costs of diagnostic delay, we assumed a conservative disease prevalence estimate (0.3% *vs* 1.2%) based on previous literature [8] and expert (rheumatologist) consultation. The NASS patient population estimate of 220 000 was also used to provide an additional estimate [45]. We assumed the UK adult population in mid-2022 as reported by the Office of National Statistics [46].

### Sensitivity analysis

The NICE health technology evaluations manual sets out the parameters and how an economic analysis and probability sensitivity analysis (PSA) should be conducted [30]. To quantify the level of confidence in the analysis results, assess the impact of uncertainties around key model parameters, and increase the robustness of the results, we implemented a PSA following the NICE standard of health economics evaluation [30, 47].

PSA was undertaken by defining parameter values using distributions rather than point estimates. PSA was carried out for all input parameters of the model, considering minimum and maximum values of the parameters using the 95% CI when the data were available or varying by ±20%. The model was run 1000 times, using the Monte-Carlo simulation to generate outputs that can be stored and compared to check how much the results vary from the base case. Further details about parameters, values and distributions used in the PSA are shown in Supplementary Table S1, available at *Rheumatology* online.

## Results

The estimated cumulative cost of an 8.5-year time to diagnosis per person living with axial SpA (symptom onset at age 26 years) is £193 512 (95% CI: 108 770–306 789). Table 1 presents the annual costs related to the health-care system, out-of-pocket expenses, and productivity losses of delayed axial SpA diagnosis. The PSA results were consistent with the deterministic results (base case), with an average cumulative cost per patient of £196 572 (95% CI: 111 830–309 850).

**Table 1.** Base case and probabilistic sensitivity analysis results of delayed axial SpA diagnosis (8.5-year time to diagnosis) classified by cost category

Cost per category	Base case (%)	PSA (%)
**Health-care system, £ (95% CI)**	£7032.74	£7048.38
	(5549.66–9285.47)	(5565.30–9301.11)
	(3.5%)	(3.6%)
**Out-of-pocket expenses, £ (95% CI)**	£60 563.04	£67 875.70
	(32 561.78–115 978.53)	(39 874.44–123 291.20)
	(31.3%)	(34.5%)
**Productivity losses, £ (95% CI)**	£125 916.27	£121 648.16
	(70 658.27–181 525.29)	(66 390.16–177 257.20)
	(65.1%)	(61.9%)
**Total, £ (95% CI)**	£193 512.04	£196 572.24
	(108 769.71–306 789.30)	(111 829.90–309 849.50)

PSA: probabilistic sensitivity analysis.

Supplementary Fig. S1, available at *Rheumatology* online, presents the average cost per year per patient and cumulative lifetime costs, and the average annual cost by decade of the patients’ age. Fig. S1 shows the average total cost per year for different ages of symptom onset. For example, the first eight values (bars) represent the average total cost of an 8-year time to diagnosis for people whose symptom onset began at 26 years. The costs per year decrease as more people from the initial cohort/cycle are diagnosed over time (or die), thereby leaving the model.

According to the analysis by gender, men had a higher total cost than women (£187 462 *vs* £182 960—Table 2). This difference in total cost was led by the difference in productivity losses, which was higher among men (see Table 2).

**Table 2.** Base case and probabilistic sensitivity analysis results of delayed axial SpA diagnosis (8.5-year time to diagnosis) classified by gender and cost category

*Female*		
Cost per category	Base case (%)	PSA (%)
**Health-care system, £ (95% CI)**	£8556.80	£8571.38
	(6268.82–11 981.16)	(6283.40–11 995.74)
	(4.7%)	(4.7%)
**Out-of-pocket expenses, £ (95% CI)**	£54 734.77	£58 650.46
	(26 960.32–106 102.19)	(30 876.01–110 017.88)
	(29.9%)	(32.2%)
**Productivity losses, £ (95% CI)**	£119 668.90	£115 039.41
	(63 264.07–178 269.09)	(58 634.58–173 639.60)
	(65.4%)	(63.1%)
**Total, £ (95% CI)**	£182 960.47	£182 261.25
	(96 493.20–296 352.44)	(95 793.99–295 653.22)

***Male***		
**Cost per category (95% CI)**	**Base case (%)**	**PSA (%)**
**Health-care system, £ (95% CI)**	£7775.60	£7812.16
	(5373.88–11 490.44)	(5388.46–11 505.02)
	(4.7%)	(4.2%)
**Out-of-pocket expenses, £ (95% CI)**	£53 255.48	£55 814.57
	(25 151.42–98 164.18)	(27 710.51–100 723.27)
	(28.4%)	(30.1%)
**Productivity losses, £ (95% CI)**	£126 431.03	£121 778.53
	(68 867.49–186 270.02)	(64 214.99–181 617.52)
	(67.4%)	(65.7%)
**Total, £ (95% CI)**	£187 462.11	£185 405.25
	(99 392.79–295 924.63)	(97 313.96–293 845.81)

PSA: probabilistic sensitivity analysis.

We extrapolated the results of the economic analysis to calculate the total annual cost for the entire UK population, using the cost per year of late diagnosis of axial SpA. We assumed the total number of patients in the UK was as estimated by NASS (220 000 patients) [45], and an axial SpA prevalence of 0.3% (ASAS classification criteria) and 1.2% (ESSG classification criteria) [8] to estimate the nationwide total annual cost. Assuming the axial SpA population in the UK as estimated by NASS in 2022, the total yearly cost was approximately £4.2 billion. The total annual cost resulting from delayed axial SpA diagnosis in the UK has been estimated at between £3.1 billion and £12.5 billion, based on a prevalence of 0.3% and 1.2%, respectively. Further details are described in Table 3.

**Table 3.** Nationwide total cost of delayed axial SpA diagnosis based on prevalence estimates

Total cost	Base case	PSA
**Modelled, £ (95% CI)**	£23 104.17	£24 025.06
	(14 568.52–36 950.02)	(15 489.41–37 870.91)
**NASS axial SpA pop** ^a^ **, £ (95% CI)**	£4 236 794 775	£4 431 645 007
	(2 703 455 437–6 595 614 347)	(2 898 305 669–6 790 464 579)
**UK adult pop** ^b^ **(0.3% prevalence), £ (95% CI)**	£3 115 805 703	£3 259 101 637
	(1 988 163 769–4 850 518 822)	(2 131 459 703–4 993 814 756)
**UK adult pop** ^b^ **(1.2% prevalence), £ (95% CI)**	£12 463 222 813	£13 036 406 549
	(7 952 655 079–19 402 075 288)	(8 525 838 815–19 975 259 024)

83.4% remaining undiagnosed per year.

PSA: probabilistic sensitivity analysis; NASS: National Axial Spondyloarthritis Society; pop: population.

a NASS patient population estimate: 220 000 [45].

b UK adult population (2022): 53 930 490 [46].

## Discussion

Many UK studies have demonstrated the high cost of axial SpA post diagnosis [15, 16, 19]. However, the time to diagnosis in the UK is estimated at 8.5 years [9], and this period is associated with a significant clinical, humanistic and economic burden [13]. Here, we estimated that the cumulative costs of delayed diagnosis per person living with axial SpA was £193 512. We assumed that, after diagnosis, the patient will receive effective treatment, and their condition will be kept under control. In particular, the costs were led by productivity losses (£125 916) and out-of-pocket expenses (£60 563). The total annual cost resulting from delayed axial SpA diagnosis in the UK was estimated at £3.1 billion and £12.5 billion, based on a prevalence of 0.3% and 1.2%, respectively. To the best of our knowledge, this is the first economic evaluation study estimating the annual cost of delayed axial SpA diagnosis in the UK, adopting both NHS (UK) and societal perspectives.

Our results indicate that the cost of delayed axial SpA diagnosis is significant, with the majority falling on the patient and society. Indeed, the results suggest that by delaying the diagnosis of axial SpA in a mixed cohort of people presumed to be living with axial SpA and with an age of symptom onset of 26 years, productivity losses and out-of-pocket expenses represent 65.1% and 31.3% of the total annual costs, respectively. These results corroborate with post-diagnosis economic studies (mainly radiographic axial SpA), whereby total costs were found to be dominated by productivity losses, informal care by family and/or out-of-pocket expenses, instead of direct health-care costs [15, 17, 19, 20, 21]. Our model did not estimate costs beyond the point of diagnosis. However, given that productivity losses account for a large proportion of the total costs, and delayed diagnosis is associated with increased risk of becoming work disabled [48], we posit that reducing time to diagnosis will lead to overall costs savings and better patient outcomes. Future models could consider both pre- and post-diagnosis costs.

Data pertaining to the costs of diagnostic delay are sparse, posing a challenge to between-study comparison. However, a recent study estimated that, in the 3 years prior to a SpA diagnosis, the cost to examine and manage 38 232 new patients in Italy between 2010 and 2013 was over €5.4 million [24]. This study highlights a significant economic burden pre-diagnosis, but it is limited in not considering indirect costs (e.g. productivity losses and out-of-pocket expenses) and in utilizing International Classification of Diseases (ICD) codes that include diagnoses such as spinal enthesopathy [24]. Other studies have demonstrated that those with longer time to diagnosis have worse economic outcomes than those with shorter time to diagnosis [49, 50]. Our study is the first to comprehensively model the potential costs of a lengthy time to diagnosis in axial SpA for the health-care system, individuals with axial SpA, and society. Strategies such as HCP education, dispelling axial SpA myths, public awareness campaigns, establishing local referral pathways and utilizing digital solutions in primary care, may be important for reducing diagnostic delay [11], and in turn the economic burden, prior to a diagnosis.

An interesting finding was the gender difference observed in costs prior to a diagnosis of axial SpA. Our results indicate than men have greater total costs, led by a higher burden of productivity losses. A possible reason for this difference might be work participation and pay inequalities in the UK. However, there may be *hidden* costs in the productivity losses, potentially due to child-care needs when managing a flare. It should be noted that health-care costs and out-of-pocket expenses were marginally higher in women than in men. Women report longer diagnostic delays than men, which may be due to physician bias or gender differences in disease manifestation [22]. Previous research has also indicated that women report more visits to HCPs prior to a diagnosis [22] and a greater use of alternative therapies [22, 51]. Such findings may be consistent with increased health-care costs and out-of-pocket expenses for women. More research is needed determine the unmet needs of females and the potential economic inequalities.

### Model strengths and limitations

This is the first economic model developed to estimate the annual cost of delayed axial SpA diagnosis in the UK, adopting both NHS and societal perspectives. Most studies have assessed the economic burden of those diagnosed with axial SpA [16–21], overlooking the cost accumulated before diagnosis. A Markov model is an efficient, parsimonious, and accurate method for modelling disease or process progression over time [52]. Notably, NICE has previously used Markov modelling to estimate the costs and utility of being correctly identified as having SpA and having the disease missed [32]. Our model incorporated input parameters derived from a diverse range of data sources and considered potential productivity losses and out-of-pocket expenses, which have often been neglected in the extant literature. Additionally, our model differentiated economic burden by gender and adds to the growing body of research highlighting gender differences in the patient journey to diagnosis [22].

Our study has some limitations. The model was based on limited available data and requires validation using real-world data to ensure its accuracy and reliability. The lack of prolonged follow-up data and existing literature on physiotherapy visits necessitated the extrapolating of survey data over a 1-year period to estimate physiotherapy costs across the entire duration of the model. This approach may have led to an overestimation of costs and should be addressed in future economic models. Further, costs pertaining to absenteeism, presenteeism and staff turnover were calculated based on the average annual costs of public and private sector employees [42]. Data from a national registry would have provided a more precise reflection of these parameters.

The estimated time to diagnosis is based on data from a decade ago [9]. Despite encouraging findings from a recent UK study reporting an average time to diagnosis of between 5 and 6 years [53], the sample is small, derived from two specialist centres, and may thus not reflect the national average. The model also used self-report data from individuals completing the NASS *State of the Nation Survey* (2016) [31], which may be subject to recall bias and may thus somewhat hinder the validity of the results. For example, the frequency of OTC medication use was based on data from a survey completed by individuals who self-reported a diagnosis of axial SpA and who may underestimate the use of OTC medication as they are currently receiving more advanced and targeted therapies. There remains uncertainty regarding referral strategies; however, our model used high-quality research to gather diagnostic sensitivity and specificity [34].

Despite every effort to include the most pertinent costs related to diagnostic delay, some costs (e.g. caregiver costs or talking therapies for mental health) could not be included within the model due to a lack of available data, and therefore the economic burden may be higher than estimated. This study did not stratify costs by disease severity, which is a potential area for future research. Additionally, including time-dependent probabilities or a patient-level simulation relying on risk equations that account for age could enhance future research. The data availability and the project scope did not allow for further investigation.

## Conclusion

Delayed axial SpA diagnosis carries significant societal costs due to productivity losses. The financial burden for people living with axial SpA (out-of-pocket expenses) is also substantial. Our findings will assist national decision-makers to understand the substantial economic burden of diagnostic delay. Early diagnosis and treatment could offer significant benefits to the patient and potentially reduce productivity losses; however, further research is needed to evaluate the long-term health economic impact. Improving public and health-care community awareness of the signs and symptoms of axial SpA and establishing local referral pathways may be important for reducing diagnostic delay and relieving some of the associated economic burden.

## Supplementary Material

keaf226_Supplementary_Data

## Data Availability

The data underlying this article may be shared on request to the corresponding author.
