# Supplementary material for: What is the economic burden of delayed axial spondyloarthritis diagnosis in the UK?
Source: Rheumatology (Oxford). 2025 Apr 25;64(9):4913–20. doi: 10.1093/rheumatology/keaf226 (PMC12407237; doi:10.1093/rheumatology/keaf226)
Supplement: keaf226_Supplementary_Data [file keaf226_supplementary_data.zip › keaf226_Supplementary_Data/rhe-24-2732-File002.docx]

**Table S1.** Input parameters

| Model parameters | Value | 95% LCI | 95% UCI | Distribution | Source |
| --- | --- | --- | --- | --- | --- |
| Global parameters | | | | | |
| Population starting age (years) | 26 | - | - | - | [1] |
| Discount rate (%) | 3.5 | - | - | - | [2] |
| Sex (% male) | 64 | 57.9 | 70.1 | Beta | [3, 4] |
| Resource use – Physiotherapist in the past 12 months | | | | | |
| Hospital-based specialist rheumatology physiotherapist | 0.654 | 0.626 | 0.682 | Dirichlet | [5] |
| NHS community-based physiotherapist | 0.150 | 0.129 | 0.171 | Dirichlet | [5] |
| Private community-based physiotherapist | 0.108 | 0.090 | 0.127 | Dirichlet | [5] |
| NASS group physiotherapist | 0.287 | 0.261 | 0.315 | Dirichlet | [5] |
| Other | 0.057 | 0.044 | 0.072 | Dirichlet | [5] |
| Frequency/proportion - Physiotherapist visits | | | | | |
| People visiting a physiotherapist more than once a week | 0.040 | 0.023 | 0.061 | Dirichlet | Experts’ opinion, [5] |
| People visiting a physiotherapist weekly | 0.313 | 0.268 | 0.359 | Dirichlet | Experts’ opinion, [5] |
| People visiting a physiotherapist fortnightly | 0.213 | 0.174 | 0.254 | Dirichlet | Experts’ opinion, [5] |
| People visiting a physiotherapist monthly | 0.120 | 0.090 | 0.154 | Dirichlet | Experts’ opinion, [5] |
| People visiting a physiotherapist less than once a month | 0.315 | 0.270 | 0.361 | Dirichlet | Experts’ opinion, [5] |
| GP visits per year | 6.92 | 5.56 | 8.32 | Lognormal | [6] |
| A&E visits | 0.870 | 0.814 | 0.916 | Lognormal | Experts’ opinion, [6] |
| Delay between the development of symptoms and presentation to NHS - Proportion of people presenting in each period | | | | | |
| 0-3 months | 0.240 | 0.201 | 0.281 | Dirichlet | [5] |
| 3-6 months | 0.122 | 0.093 | 0.154 | Dirichlet | [5] |
| 6 months – 1 year | 0.187 | 0.151 | 0.225 | Dirichlet | [5] |
| 1 year – 5 years | 0.237 | 0.198 | 0.278 | Dirichlet | [5] |
| 5 years+ | 0.214 | 0.177 | 0.254 | Dirichlet | [5] |
| Proportion of people diagnosed in each period | | | | | |
| 0-3 months | 0.091 | 0.066 | 0.120 | Dirichlet | [5] |
| 3-6 months | 0.105 | 0.078 | 0.135 | Dirichlet | [5] |
| 6 months – 1 year | 0.107 | 0.080 | 0.138 | Dirichlet | [5] |
| 1 year – 3 years | 0.158 | 0.125 | 0.193 | Dirichlet | [5] |
| 3 years – 5 years | 0.080 | 0.056 | 0.107 | Dirichlet | [5] |
| 5 years – 7 years | 0.066 | 0.045 | 0.091 | Dirichlet | [5] |
| 7 years – 10 years | 0.087 | 0.062 | 0.115 | Dirichlet | [5] |
| 10 years+ | 0.306 | 0.264 | 0.350 | Dirichlet | [5] |
| Proportions – Comorbidities | | | | | |
| Comorbidities presentation at 3-months | 0.050 | 0.039 | 0.050 | Beta | [7] |
| Chronic back pain | 0.050 | 0.039 | 0.053 | Beta | [7] |
| Uveitis | 0.120 | 0.116 | 0.122 | Beta | [7] |
| Psoriasis | 0.043 | 0.030 | 0.045 | Beta | [7] |
| Inflammatory bowel disease | 0.026 | 0.021 | 0.028 | Beta | [7] |
| Depression (delay ≥7 years) | 0.043 | 0.039 | 0.045 | Beta | [8] |
| Depression (delay <7 years) | 0.024 | 0.018 | 0.027 | Beta | [8] |
| Proportions - Out of Pocket Expenses | | | | | |
| People who had visited a chiropractor | 0.433 | 0.402 | 0.464 | Dirichlet | [5] |
| People visiting a chiropractor more than once a week | 0.217 | 0.198 | 0.282 | Dirichlet | [5] |
| People visiting a chiropractor weekly | 0.445 | 0.423 | 0.478 | Dirichlet | [5] |
| People visiting a chiropractor fortnightly | 0.213 | 0.174 | 0.254 | Dirichlet | [5] |
| People visiting a chiropractor monthly | 0.120 | 0.090 | 0.154 | Dirichlet | [5] |
| People visiting a chiropractor less than once a month | 0.315 | 0.270 | 0.361 | Dirichlet | [5] |
| People who had visited an osteopath | 0.359 | 0.329 | 0.390 | Dirichlet | [5] |
| People visiting an osteopath more than once a week | 0.009 | 0.002 | 0.022 | Dirichlet | [5] |
| People visiting an osteopath weekly | 0.336 | 0.286 | 0.389 | Dirichlet | [5] |
| People visiting an osteopath fortnightly | 0.216 | 0.173 | 0.262 | Dirichlet | [5] |
| People visiting an osteopath monthly | 0.133 | 0.098 | 0.172 | Dirichlet | [5] |
| People visiting an osteopath less than once a month | 0.306 | 0.257 | 0.357 | Dirichlet | [5] |
| Proportion - Medication | | | | | |
| NSAIDs e.g. ibuprofen, Anadin extra etc | 0.240 | 0.224 | 0.256 | Dirichlet | [5] |
| Topical anti-inflammatory gels, creams, or sprays | 0.220 | 0.205 | 0.235 | Dirichlet | [5] |
| Rubefacients: heat rubs, sprays, and gels) e.g. Deep Heat Rub | 0.120 | 0.108 | 0.132 | Dirichlet | [5] |
| Paracetamol | 0.430 | 0.412 | 0.448 | Dirichlet | [5] |
| Aspirin | 0.050 | 0.042 | 0.058 | Dirichlet | [5] |
| Co-codamol e.g. Solpadeine | 0.100 | 0.089 | 0.111 | Dirichlet | [5] |
| Glucosamine and/or Chondroitin | 0.060 | 0.052 | 0.069 | Dirichlet | [5] |
| Natural medicines (herbal remedies) | 0.090 | 0.080 | 0.101 | Dirichlet | [5] |
| Others | 0.060 | 0.052 | 0.069 | Dirichlet | [5] |
| Proportion - Employment status | | | | | |
| Full-time | 0.352 | 0.330 | 0.374 | Dirichlet | [5] |
| Part-time | 0.166 | 0.149 | 0.184 | Dirichlet | [5] |
| No employment (in education) | 0.008 | 0.005 | 0.013 | Dirichlet | [5] |
| No employment (full-time parent) | 0.020 | 0.014 | 0.027 | Dirichlet | [5] |
| No employment (retired) | 0.290 | 0.269 | 0.311 | Dirichlet | [5] |
| No employment (due to health issues) | 0.164 | 0.147 | 0.181 | Dirichlet | [5] |
| Proportion - Impact of AS on employment | | | | | |
| No impact | 0.086 | 0.073 | 0.099 | Dirichlet | [5] |
| Work fewer hours | 0.098 | 0.084 | 0.112 | Dirichlet | [5] |
| Go to work when not well | 0.274 | 0.253 | 0.295 | Dirichlet | [5] |
| Do less physical work | 0.145 | 0.129 | 0.161 | Dirichlet | [5] |
| Decreased job satisfaction | 0.104 | 0.090 | 0.118 | Dirichlet | [5] |
| Not preferred job | 0.053 | 0.043 | 0.064 | Dirichlet | [5] |
| Job is not the best use of skills | 0.040 | 0.032 | 0.050 | Dirichlet | [5] |
| Had to change occupation | 0.082 | 0.070 | 0.096 | Dirichlet | [5] |
| Left the job | 0.119 | 0.104 | 0.134 | Dirichlet | [5] |
| Proportion - Early Retirement | | | | | |
| Patients granted early retirement | 0.001 | 0.000 | 0.002 | Beta | [9] |
| Early retirement below 30 per delay period | 0.068 | 0.062 | 0.074 | Beta | [9] |
| Early retirement 40-50 per delay period | 0.298 | 0.287 | 0.354 | Beta | [9] |
| Early retirement above 50 per delay period | 0.634 | 0.584 | 0.659 | Beta | [9] |
| Diagnostic strategy – Sensitivity and Specificity | | | | | |
| Van Hoeven et al. (2015) (Sensitivity) | 0.926 | 0.866 | 0.970 | Beta | [10] |
| Van Hoeven et al. (2015) (Specificity) | 0.390 | 0.348 | 0.434 | Beta | [10] |
| Costs - Hospital Physiotherapist | | | | | |
| Physiotherapist specialist (Band 6) | £52.00 | £46.80 | £57.20 | Gamma | [11] |
| Physiotherapist specialist (advanced) (Band 7) | £63.00 | £56.70 | £69.30 | Gamma | [11] |
| Physiotherapist principal (Band 8) | £72.00 | £64.80 | £79.20 | Gamma | [11] |
| Physiotherapist consultant (Band 8b) | £85.00 | £76.50 | £93.50 | Gamma | [11] |
| Costs - Community Physiotherapist | | | | | |
| Physiotherapist (Band 5) | £41.00 | £36.90 | £45.10 | Gamma | [11] |
| Physiotherapist specialist (Band 6) | £54.00 | £48.60 | £59.40 | Gamma | [11] |
| Physiotherapist specialist (advanced) (Band 7) | £65.00 | £58.50 | £71.50 | Gamma | [11] |
| Physiotherapist principal (Band 8) | £75.00 | £67.50 | £82.50 | Gamma | [11] |
| Physiotherapist consultant (Band 8b) | £88.00 | £79.20 | £96.80 | Gamma | [11] |
| Costs - GP and A&E |  |  |  |  |  |
| GP Costs per Visit (15 min consultation) | £63.82 | £57.44 | £70.20 | Gamma | [11] |
| A&E cost per visit | £182.19 | £163.97 | £200.41 | Gamma | [12] |
| Admin costs per patient per consultation | £24.58 | £22.12 | £27.04 | Gamma | [12] |
| Cost - Annual Comorbidities Treatment | | | | | |
| Chronic back pain | £917.09 | £881.69 | £935.37 | Gamma | [12] |
| Uveitis | £3,123.05 | £3,067.87 | £3,183.16 | Gamma | [13] |
| Psoriasis | £3,007.31 | £2,889.43 | £3,123.13 | Gamma | [12] |
| Inflammatory bowel disease | £3,323.93 | £3,267.54 | £3,486.25 | Gamma | [12] |
| Depression | £1,857.62 | £1,679.34 | £2,004.54 | Gamma | [14] |
| Cost – Chiropractic Visit | | | | | |
| Chiropractic cost per visit | £55.00 | £50.00 | £60.00 | Gamma | [15] |
| Cost - Osteopath Visit | | | | | |
| Osteopath cost per visit | £47.50 | £42.00 | £52.50 | Gamma | [16] |
| Over the counter medication costs (3 months) per person | | | | | |
| NSAIDs e.g. ibuprofen, Anadin extra etc | £6.49 | £4.11 | £7.84 | Gamma | [17, 18], Boots UK Ltd |
| Topical anti-inflammatory gels, creams, or sprays | £5.95 | £4.79 | £6.39 | Gamma | [17, 18], Boots UK Ltd |
| Rubefacients: heat rubs, sprays, and gels) e.g. Deep Heat Rub | £4.60 | £1.76 | £6.01 | Gamma | [17, 18], Boots UK Ltd |
| Paracetamol | £7.20 | £7.08 | £7.44 | Gamma | [17, 18], Boots UK Ltd |
| Aspirin | £0.68 | £0.45 | £0.78 | Gamma | [17, 18], Boots UK Ltd |
| Co-codamol e.g. Solpadeine | £2.70 | £2.49 | £2.78 | Gamma | [17, 18], Boots UK Ltd |
| Glucosamine and/or Chondroitin | £0.32 | £0.29 | £0.44 | Gamma | [17, 18], Boots UK Ltd |
| Natural medicines (herbal remedies) | £1.07 | £1.01 | £1.58 | Gamma | [17, 18], Boots UK Ltd |
| Others | £1.07 | £0.92 | £1.12 | Gamma | [17, 18], Boots UK Ltd |
| Cost - early retirement | | | | | |
| Early retirement cost per patient per quarter | £2,026.75 | £1,998.69 | £2,103.22 | Gamma | [6] |
| Cost - Unpaid care | | | | | |
| Cost of unpaid assistance at mean wage (3 months Age < 50) | £490.00 | £488.59 | £493.71 | Gamma | [6] |
| Cost of unpaid assistance for health care visits at mean wage (3 months Age < 50) | £38.27 | £36.79 | £39.28 | Gamma | [6] |
| Cost of unpaid assistance at mean wage (3 months Age > 50) | £1,041.45 | £982.43 | £1,119.94 | Gamma | [6] |
| Cost of unpaid assistance for health care visits at mean wage (3 months Age > 50) | £63.41 | £61.15 | £64.00 | Gamma | [6] |
| Cost - Gross median wage (per hour) | | | | | |
| Full-time – All | £15.65 | £9.29 | £19.63 | Gamma | [19] |
| Part-time – All | £10.64 | £6.89 | £15.88 | Gamma | [19] |
| Full-time – Male | £16.25 | £13.08 | £19.56 | Gamma | [19] |
| Part-time – Male | £10.45 | £4.73 | £13.78 | Gamma | [19] |
| Full-time – Female | £14.87 | £9.44 | £18.82 | Gamma | [19] |
| Part-time – Female | £10.71 | £5.82 | £12.66 | Gamma | [19] |
| Cost – Presenteeism, absenteeism, and staff turnover | | | | | |
| Presenteeism | £1,685.27 | £1,516.50 | £1,853.50 | Gamma | [20] |
| Absenteeism | £427.99 | £422.73 | £431.27 | Gamma | [20] |
| Staff turnover | £318.06 | £314.82 | £321.18 | Gamma | [20] |

*Note*. NHS, National Health Service; NASS, National Axial Spondyloarthritis Society; GP, general practitioner; A&E, Accident and Emergency; NSAID, Non-Steroidal Anti-Inflammatory Drug; AS, ankylosing spondylitis.

**References**

[1] Boel A, López-Medina C, van der Heijde DMFM, van Gaalen FA. Age at onset in axial spondyloarthritis around the world: data from the Assessment in SpondyloArthritis international Society Peripheral Involvement in Spondyloarthritis study. Rheumatology (Oxford) 2022;61(4):1468–75. doi: 10.1093/rheumatology/keab544.

[2] National Institute for Health and Care Excellence (NICE). NICE health technology evaluations: the manual [Internet]. [cited 2024 Apr 24]. Available from: <https://www.nice.org.uk/process/pmg36/chapter/introduction-to-health-technology-evaluation>

[3] National Institute for Health and Care Excellence (NICE). 2017 Spondyloarthritis in over 16s: diagnosis and management [Internet]. [cited 2024 Apr 10]. Available from: <https://www.nice.org.uk/guidance/ng65>

[4] Rudwaleit M, Haibel H, Baraliakos X, Listing J, Märker‐Hermann E, Zeidler H, et al. The early disease stage in axial spondylarthritis: results from the German spondyloarthritis inception cohort. Arthritis Rheum 2009;60(3):717–27. doi: 10.1002/art.24483.

[5] Derakhshan MH, Pathak H, Cook D, Dickinson S, Siebert S, Gaffney K, et al. Services for spondyloarthritis: a survey of patients and rheumatologists. Rheumatology (Oxford) 2018;57(6):987-96. doi: 10.1093/rheumatology/kex518.

[6] Cooksey R, Husain MJ, Brophy S, Davies H, Rahman MA, Atkinson MD, et al. The cost of ankylosing spondylitis in the UK using linked routine and patient-reported survey data. PLoS One 2015;10(7):e0126105. doi: [10.1371/journal.pone.0126105](https://doi.org/10.1371%2Fjournal.pone.0126105).

[7] Martindale J, Shukla R, Goodacre J. The impact of ankylosing spondylitis/axial spondyloarthritis on work productivity. Best Pract Res Clin Rheumatol 2015;29(3):512–23. doi: [10.1016/j.berh.2015.04.002](https://doi.org/10.1016/j.berh.2015.04.002).

[8] Fitzgerald G, Gallagher P, O’Sullivan C, O’Rourke K, Sheehy C, Stafford F, et al. 112. Delayed diagnosis of axial spondyloarthropathy is associated with a higher prevalence of depression. Rheumatology 2017;56(Suppl_2):kex062.112. doi: 10.1093/rheumatology/kex062.112.

[9] Cobilinschi C, Ionescu R, Opris-Belinski D. AB1082 Impact of ankylosing spondylitis versus non-radiographic spondyloarthritis on early retirement. Ann Rheum Dis 2017;76:1433-4. doi: 10.1136/annrheumdis-2017-eular.5352.

[10] Van Hoeven L, Koes BW, Hazes JM, Weel AE. Evaluating the ASAS recommendations for early referral of axial spondyloarthritis in patients with chronic low back pain; is one parameter present sufficient for primary care practice? Ann Rheum Dis 2015;74(12):e68. doi: 10.1136/annrheumdis-2015-208547.

[11] Jones KC, Burns A. Unit Costs of Health and Social Care 2021 [Internet]. Personal Social Services Research Unit, University of Kent, Canterbury. 2021 [cited 2024 Apr 30]. Available from: <https://kar.kent.ac.uk/id/eprint/92342>. doi: 10.22024/UniKent/01.02.92342.

[12] National Health Service. 2019/20 National Cost Collection Data Publication [Internet]. 2021 [cited 2024 Apr 30]. Available from: <https://www.england.nhs.uk/publication/2019-20-national-cost-collection-data-publication/>

[13] Ocular Immunology and Uveitis Foundation – Justus A, Foster SC. Cost of Care of Patients with Uveitis [Internet]. [cited 2024 Apr 24]. Available from: <https://uveitis.org/cost-care-patients-uveitis/>

[14] Morriss R, Xydopoulos G, Craven M, Price L, Fordham R. Clinical effectiveness and cost minimisation model of Alpha-Stim cranial electrotherapy stimulation in treatment seeking patients with moderate to severe generalised anxiety disorder. J Affect Disord 2019;253:426–37. doi: 10.1016/j.jad.2019.04.020.

[15] National Health Service. Chiropractic – Overview [Internet]. [cited 2022 Mar 30]. Available from: <https://www.nhs.uk/conditions/chiropractic/>

[16] National Health Service. Osteopathy – Overview [Internet]. [cited 2022 Mar 30]. Available from: https://www.nhs.uk/conditions/osteopathy/

[17] Joint Formulary Committee. British National Formulary [Internet]. London: BMJ and Pharmaceutical Press; 2022 [cited 2024 Apr 30]. Available from: <https://bnf.nice.org.uk>.

[18] NHS Business Services Authority. NHS Prescription Prepayment Certificates (PPCs) | NHSBSA. 2022. [Internet]. [cited 2024 May 1]. Available from: <https://www.nhsbsa.nhs.uk/help-nhs-prescription-costs/nhs-prescription-prepayment-certificate-ppc>

[19] Office for National Statistics (ONS). Annual Survey of Hours and Earnings (ASHE) [Internet]. [cited 2024 Apr 30]. Available from: <https://www.ons.gov.uk/surveys/informationforbusinesses/businesssurveys/annualsurveyofhoursandearningsashe>

[20] Deloitte. Mental health and employers - Refreshing the case for investment [Internet]. 2020 [cited 2024 Apr 30]. Available from: <https://www2.deloitte.com/content/dam/Deloitte/uk/Documents/consultancy/deloitte-uk-mental-health-and-employers.pdf>
