# Supplementary material for: What is the economic burden of delayed axial spondyloarthritis diagnosis in the UK?
Source: Rheumatology (Oxford). 2025 Apr 25;64(9):4913–20. doi: 10.1093/rheumatology/keaf226 (PMC12407237; doi:10.1093/rheumatology/keaf226)

**Figure S1.** Cost per year and cumulative costs (lifetime)

Average cost per year per decade of patients’ age

| **Decade of patients’ age** | **Cost** |
| --- | --- |
| 26 - 35 | £22,318.88 |
| 36 - 45 | £15,927.10 |
| 46 - 55 | £11,330.91 |
| 56 - 65 | £7,861.40 |
| 66 - 75 | £4,144.00 |
| 76 - 85 | £1,751.82 |
| 86+ | £205.88 |


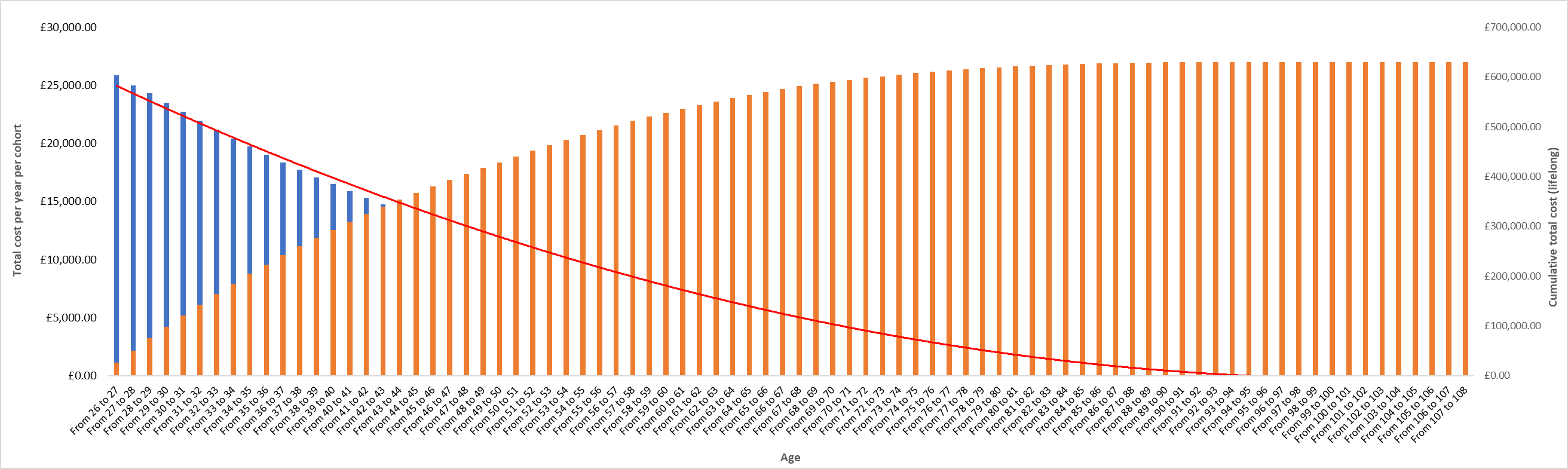

Supplement: keaf226_Supplementary_Data [file keaf226_supplementary_data.zip › keaf226_Supplementary_Data/rhe-24-2732-File003.docx]
